# Nitric oxide, antioxidants and prooxidants in plant defence responses

**DOI:** 10.3389/fpls.2013.00419

**Published:** 2013-10-29

**Authors:** Felicitas Groß, Jörg Durner, Frank Gaupels

**Affiliations:** German Research Center for Environmental Health, Institute of Biochemical Plant Pathology, Helmholtz-Zentrum MünchenMunich, Germany

**Keywords:** nitric oxide, reactive oxygen species, signaling, peroxynitrite, glutathione, ascorbate, antioxidant system, programmed cell death

## Abstract

In plant cells the free radical nitric oxide (NO) interacts both with anti- as well as prooxidants. This review provides a short survey of the central roles of ascorbate and glutathione—the latter alone or in conjunction with S-nitrosoglutathione reductase—in controlling NO bioavailability. Other major topics include the regulation of antioxidant enzymes by NO and the interplay between NO and reactive oxygen species (ROS). Under stress conditions NO regulates antioxidant enzymes at the level of activity and gene expression, which can cause either enhancement or reduction of the cellular redox status. For instance chronic NO production during salt stress induced the antioxidant system thereby increasing salt tolerance in various plants. In contrast, rapid NO accumulation in response to strong stress stimuli was occasionally linked to inhibition of antioxidant enzymes and a subsequent rise in hydrogen peroxide levels. Moreover, during incompatible *Arabidopsis thaliana*-*Pseudomonas syringae* interactions ROS burst and cell death progression were shown to be terminated by S-nitrosylation-triggered inhibition of NADPH oxidases, further highlighting the multiple roles of NO during redox-signaling. In chemical reactions between NO and ROS reactive nitrogen species (RNS) arise with characteristics different from their precursors. Recently, peroxynitrite formed by the reaction of NO with superoxide has attracted much attention. We will describe putative functions of this molecule and other NO derivatives in plant cells. Non-symbiotic hemoglobins (nsHb) were proposed to act in NO degradation. Additionally, like other oxidases nsHb is also capable of catalyzing protein nitration through a nitrite- and hydrogen peroxide-dependent process. The physiological significance of the described findings under abiotic and biotic stress conditions will be discussed with a special emphasis on pathogen-induced programmed cell death (PCD).

## Introduction

Exposure of plants to abiotic and biotic stress can cause a deregulation, over-flow or even disruption of electron transport chains (ETC) in mitochondria and chloroplasts. Under these conditions molecular oxygen (O_2_) acts as an electron acceptor giving rise to the accumulation of reactive oxygen species (ROS). Singlet oxygen (^1^O_2_), the hydroxyl radical (OH), the superoxide radical (O^−^_2_) and hydrogen peroxide (H_2_O_2_) are all strongly oxidizing compounds and therefore potentially harmful for cell integrity. Among them, H_2_O_2_ is the most stable ROS being formed in the reaction of ^1^O_2_ with O^−^_2_ and as a product of spontaneous dismutation of O^−^_2_ (Foyer and Noctor, 2009).

**Figure 1:**
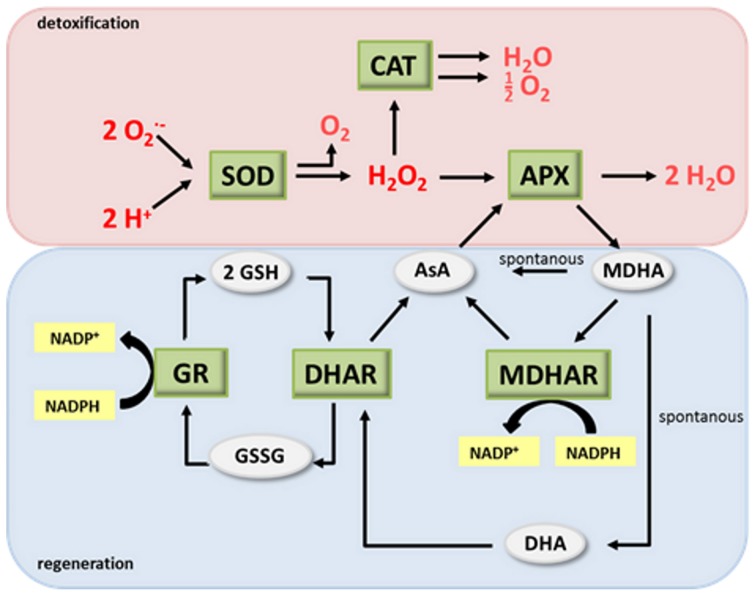
**The antioxidant system. (modified after Buchanan et al., 2002)**. AsA, ascorbate; DHA, dehydroascorbate; SOD, superoxide dismutase; CAT, catalase; APX, ascorbate peroxidase; MDHA, monodehydroascorbate; MDHAR, MDHA reductase; DHAR, DHA reductase; GR, glutathione reductase; GSH glutathione; GSSG, glutathione disulphide.

During evolution, land plants have developed sophisticated measures for controlling ROS levels amongst others by the antioxidant system or—as named after their discoverers—Foyer-Halliwell-Asada cycle (Figure 1) (Buchanan et al., 2002; Foyer and Noctor, 2009). Central elements of the system are the two redox couples ascorbate (AsA)/dehydroascorbate (DHA) and glutathione (GSH)/glutathione disulfide (GSSG). In the detoxification part of the antioxidant system superoxide dismutase (SOD) converts O^−^_2_ to O_2_ and H_2_O_2_. The latter then can be degraded by catalase (CAT), ascorbate peroxidase (APX) and several other enzymes (Figure 1). In the course of H_2_O_2_ degradation by APX AsA is oxidized to monodehydroascorbate (MDHA) and DHA. AsA and GSH can also directly be oxidized by ROS, although with slower kinetics. In the regeneration pathway MDHA reductase (MDHAR), DHA reductase (DHAR) and glutathione reductase (GR) recycle the antioxidants from their oxidized back to the reduced form. MDHAR and GR use NADPH as a reducing equivalent whereas DHAR uses GSH (Figure 1).

However, apart from being toxic by-products of energy metabolism, ROS have also essential functions in primary and secondary metabolism, development, and stress responses. For instance, H_2_O_2_ acts as a signal in the regulation of stomatal closure and serves as a substrate of peroxidases during cell wall synthesis and fortification (Neill et al., 2008; O'brien et al., 2012). To date, O^−^_2_ and H_2_O_2_ are the best studied ROS, mainly because of well-established detection techniques. During signaling processes, ROS arises from the ETC but are also enzymatically produced by various peroxidases and oxidases (Foyer and Noctor, 2009; Mittler et al., 2011). Here, we will assign the term prooxidants for ROS and ROS-producing enzymes and the term antioxidants for elements of the antioxidant system. During stress signaling, the redox homeostasis of plant cells is tightly controlled. Antioxidants modulate timing and extent of ROS accumulation and additionally function as signals by their own rights. ROS levels increase either by up-regulation of prooxidant enzyme activity, (de−) regulation of electron flow or down-regulation of the antioxidant system. Redox signals are probably transduced by oxidation of proteins such as ROS-activated transcription factors and kinases (Foyer and Noctor, 2009; Mittler et al., 2011). Also other molecules including lipids and fatty acids are modified by ROS with implications for their signaling functions (Farmer and Mueller, 2013).

Similar to ROS, NO is a small redox signal with versatile chemistry. It is a relatively stable radical but rapidly reacts with other radicals including ROS (Hill et al., 2010). Products of these reactions are reactive nitrogen species (RNS) such as the nitrosonium cation (NO^+^), the nitroxyl anion (NO^−^) and higher oxides of NO including ONOO^−^, NO_2_, and N_2_O_3_. RNS have chemical properties different from their precursors and may trigger specific physiological responses. Like ROS, NO is an important messenger in many physiological processes. It is a stress signal involved in plant responses to high salt, excess light, cold, heat, ozone, UV-B and various pathogens (Leitner et al., 2009; Gaupels et al., 2011a; Mur et al., 2013). Despite the ever-growing importance of NO in plant research, only little is known about enzymatic sources and molecular receptors of NO. Best characterized is the role of NO in stomatal closure and pathogen defence (Mur et al., 2013). In both processes, NO interacts with H_2_O_2_ without exact molecular mechanisms deciphered.

The aim of this review is to summarize current knowledge on the interaction of NO with ROS and the antioxidant system in plant stress responses. We will explore how NO can chemically react with pro- and antioxidants and how NO might regulate activity and expression of pro- and antioxidant enzymes. Additionally, functions of non-symbiotic hemoglobins, SOD, GSNOR and peroxiredoxins in regulating RNS homeostasis will be discussed. The last section of this review will detail the roles of individual NO and redox messengers in signaling during stress-induced programmed cell death (PCD).

## Manipulation of the NO level has an impact on the antioxidant system

The relevance of NO in stress-induced redox signaling was repeatedly investigated by treatment of plants with NO donors before or during exposure to abiotic stress conditions (Hasanuzzaman et al., 2010; Saxena and Shekhawat, 2013). Table 1 summarizes selected literature reporting the impact of NO donor treatment on H_2_O_2_ level, antioxidants and activity of antioxidant enzymes in stressed plants. The authors studied 14 different plant species, 11 stressors, and 6 different NO donors providing a comprehensive overview of the current literature on this topic. A common effect of all stress treatments was the accumulation of H_2_O_2_ often accompanied by an increase in malondialdehyde (MDA) levels pointing to ROS-dependent oxidation of lipids. In 19 of the 23 studies activities of all or at least some of the analyzed antioxidant enzymes were up-regulated. These data suggest that stress causes accumulation of ROS, which may then trigger enhancement of the antioxidant defence system.

**Table 1:**
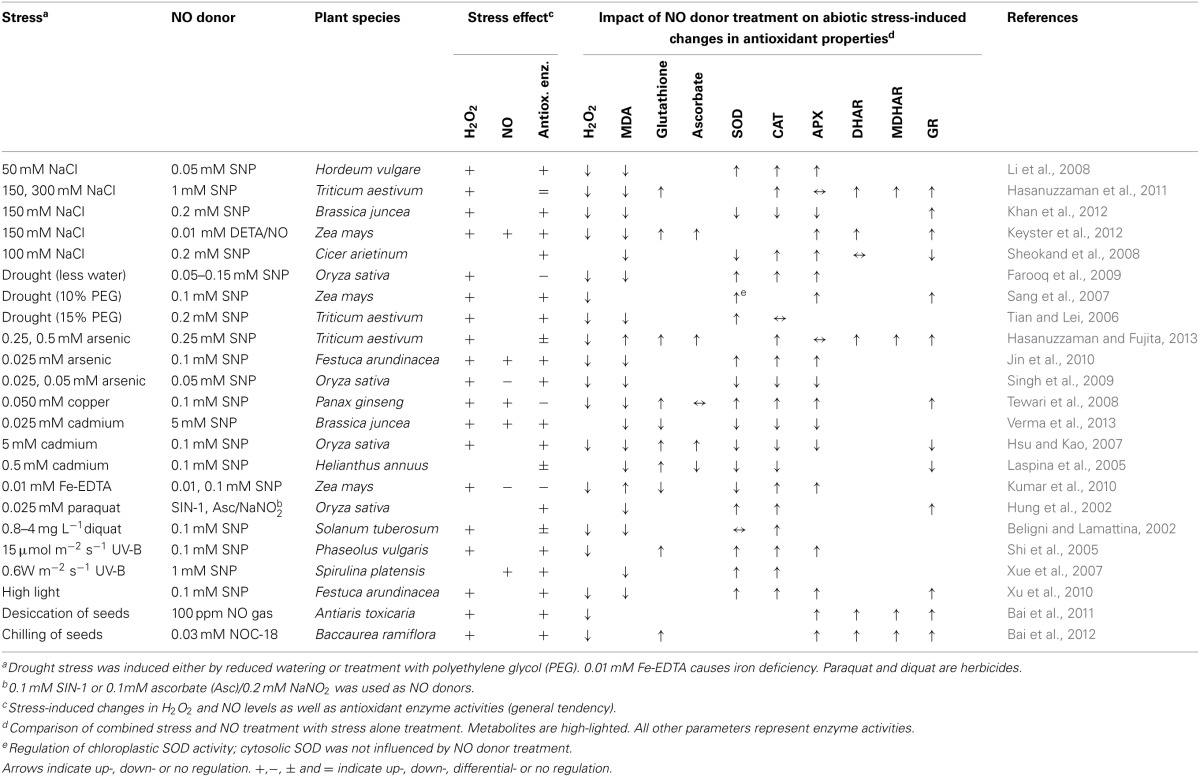
**NO donors induce stress tolerance by effecting on the antioxidant properties of plant tissues**.

^a^Drought stress was induced either by reduced watering or treatment with polyethylene glycol (PEG). 0.01 mM Fe-EDTA causes iron deficiency. Paraquat and diquat are herbicides.

^b^0.1 mM SIN-1 or 0.1mM ascorbate (Asc)/0.2 mM NaNO_2_ was used as NO donors.

^c^Stress-induced changes in H_2_O_2_ and NO levels as well as antioxidant enzyme activities (general tendency).

^d^Comparison of combined stress and NO treatment with stress alone treatment. Metabolites are high-lighted. All other parameters represent enzyme activities.

^e^Regulation of chloroplastic SOD activity; cytosolic SOD was not influenced by NO donor treatment.

Arrows indicate up-, down- or no regulation. +,−, ± and = indicate up-, down-, differential- or no regulation.

Most of the published studies demonstrated accumulation of NO under stress conditions (Hasanuzzaman et al., 2010; Saxena and Shekhawat, 2013). However, results given in Table 1 as well as other data imply that NO cannot be considered to be a general stress signal. For instance, comparing the effect of 25 μM arsenic between two studies, NO production was induced in *Festuca arundinaceae* but decreased in *Oryza sativa* (Table 1) (Singh et al., 2009; Jin et al., 2010). During plant responses to cadmium stress, NO was increased or decreased acting as inducer or inhibitor of stress tolerance, depending on plant species and experimental setup (Arasimowicz-Jelonek et al., 2011a). Moreover, iron deficiency triggered NO signaling in *Arabidopsis thaliana* (Chen et al., 2010) but repressed basal NO synthesis in *Zea mays* (Table 1) (Kumar et al., 2010). In this context it is interesting that recent studies revealed NO being a modulator rather than an essential signal in the adaptation of *A. thaliana* to iron deficiency (Meiser et al., 2011). Together, these findings demonstrate that the link between stress perception and NO signaling is seemingly rather indirect whereas stress can directly cause ROS accumulation by disturbing the mitochondrial and plastidic ETC. Further studies are needed for investigating the biological background of the observed species-specific differences in NO regulation under stress conditions. In sum, the above findings support the notion that endogenous NO is often but not always involved in stress tolerance.

Exogenous NO always improved abiotic stress tolerance concomitant with a decrease in H_2_O_2_ and MDA levels (Table 1). This held true, even when endogenous NO was down-regulated, implying that the tested NO donors do not necessarily mimic functions of NO under natural conditions. In the displayed 23 studies, NO treatments either reversed the stress-induced decline or even further amplified up-regulation of the antioxidant system. NO donors never caused a down-regulation of antioxidant enzymes as compared to untreated control plants. For instance, salt stress stimulated SOD, CAT, and APX activities, and this effect was enhanced by SNP co-treatment, whereas copper uptake repressed the same enzymes in *Panax ginseng*, which was prevented by SNP (Table 1) (Li et al., 2008; Tewari et al., 2008). Again the same enzyme activities were enhanced after arsenic poisoning of *O. sativa* but SNP application prevented this stress effect (Table 1) (Singh et al., 2009). These findings were explained by NO acting either (I) as a direct scavenger of ROS or (II) inducer of the antioxidant system. In the first case NO would take over functions of the antioxidant system and thereby prevent its activation, like e.g. in arsenic-exposed rice as described above. In the second case NO would trigger antioxidant gene expression or activate antioxidant enzymes e.g., by posttranslational modifications. Previously, NO donors were reported to repress antioxidant enzyme activities. Particularly, SNP inhibited APX and CAT, decreased GSH/GSSG ratio and induced PCD in Arabidopsis suspension cultured cells (Murgia et al., 2004a). However, the research summarized in Table 1 was focussed on investigating mechanisms of NO-mediated stress tolerance. Therefore, NO donors were probably applied in such a way as to prevent any severe stress or damage to the plants although sometimes up to 5 mM SNP was used. We will discuss later in this review the dose dependent effects of NO on the antioxidant system and cell death initiation.

A direct chemical interaction of NO with ROS is only possible if cells or plant parts are being loaded with active NO donor solution from start of the stress treatment until sampling as was the case for *Spirulina platensis* cells exposed to UV-B and SNP and *Brassica junceae* leaf discs incubated in salt and DETA/NO donors (Table 1) (Xue et al., 2007; Khan et al., 2012). In other studies, however, measurements were done after NO donors were exhausted suggesting that NO released from the donor did not have a direct influence on ROS levels but might be rather involved in the induction of signaling events controlling the cellular redox status. Farooq et al. (2010) reported that imbibition of seeds in SNP solution rendered adult rice plants more tolerant to drought stress. Hence, NO pre-treatment could induce a primed state, which prepares plants to respond more efficiently to future stress episodes (Conrath, 2011). Alternatively, NO treatment itself could impose stress to the plants acting as the priming stimulus. Exogenous NO might also induce synthesis of endogenous NO, which then can exert signaling or scavenger functions even long after the NO donor is exhausted.

NO donors can have undesired side-effects on the plant's physiology. Therefore, NO accumulating transgenic and mutant plant lines were used for assessing the involvement of NO in development and stress signaling. Transgenic *Nicotiana tabacum* and *A. thaliana* expressing the rat neuronal nitric oxide synthase (NOS) behind a 35S promoter accumulated high levels of NO concomitant with developmental defects and altered stress resistance (Chun et al., 2012; Shi et al., 2012). 35S::nNOS lines of Arabidopsis constitutively expressed pathogenesis related (PR) genes, which correlated with enhanced pathogen resistance toward virulent *Pseudomonas syringae DC3000* (Shi et al., 2012). These plants also had improved salt and drought tolerance due to reduced stomatal aperture, and were delayed in flowering. The H_2_O_2_ content was not determined, but MDA levels were found to be lowered. By comparison, nNOS-expressing tobacco showed growth retardation and constitutive inhibition of CAT, which caused an increase in H_2_O_2_ levels (Chun et al., 2012). Probably as a consequence of high NO and H_2_O_2_ levels, these plants developed spontaneous lesions, strongly elevated salicylic acid (SA) levels and PR gene expression. Reduced growth, increased oxidative stress and spontaneous lesions was not observed in nNOS expressing *A. thaliana* plants indicating that they either were less sensitive to NO or accumulated lower levels of NO than the corresponding tobacco transgenic lines.

Collectively, the discussed research argues for ROS being a general stress signal whereas NO signaling depends on the plant species and stress conditions investigated. It can be speculated that NO or the interaction between ROS and NO adds some degree of specificity to the stress signaling by ROS alone. Treatment of plants with NO donors caused a decrease in stress-induced ROS levels and a concomitant enhancement of abiotic stress tolerance. In this process NO might act as a scavenger of ROS or as a signal stimulating the antioxidant potential and/or a primed state of stress defence. Interpretation of the data is complicated by the fact that most of the studies are rather descriptive without exploring the underlying signaling cascades. Moreover, the biological significance of some observed weak effects of NO on ROS and the antioxidant system is ambiguous because slight changes in the cellular redox status could be just a stress marker.

## Sources and cellular localization of NO and ROS production

NO and certain ROS cooperate in stress signaling, which is partly independent of their respective production sites because both molecules are supposed to be mobile intra- as well as intercellularly (Foyer and Noctor, 2009; Frohlich and Durner, 2011). Therefore, apoplastic sources can contribute to NO and ROS signal transduction within the cell (Table 2). Important ROS producing enzymes are the members of the NADPH oxidase family (NOX or Respiratory burst oxidase homolog, RBOH). These plasma membrane-associated enzymes synthesize O^−^_2_ in the apoplast through transfer of electrons from NADPH to molecular oxygen (Mittler et al., 2011). A rapid ROS burst, frequently observed during plant responses to pathogen infection, is usually mediated by the NOX isoforms D and F (Torres et al., 2002). Further oxidases and cell wall-associated peroxidases are present in the apoplast but their roles in stress responses are less well-defined. In comparison to ROS only little is known about NO formation in the extracellular space (Table 2). At the acidic pH of the apoplast exogenous NO^−^_2_ was non-enzymatically reduced to NO, which was accelerated by AsA and phenolics (Bethke et al., 2004). The pathway has been investigated in the barley aleuron layer but might occur also in other tissues. A stress-induced NO burst derived from this spontaneous reaction seems only feasible if NO^−^_2_ levels could be rapidly up-regulated, which has not been observed so far. NO^−^_2_ could also be reduced to NO by a membrane-associated nitrite:NO reductase (NiNOR) as described for tobacco (Stöhr et al., 2001). However, NiNOR cannot be considered a major player in NO signaling because it is exclusively present in roots functioning in the regulation of NO^−^_3_ uptake. Copper amine oxidase 1 (CuAO1) is another candidate enzyme involved in NO synthesis (Wimalasekera et al., 2011). The *A. thaliana* cuao1 mutant is impaired in polyamine- and abscisic acid-induced NO production. The molecular background underlying this interesting phenotype is still unknown.

**Table 2:**
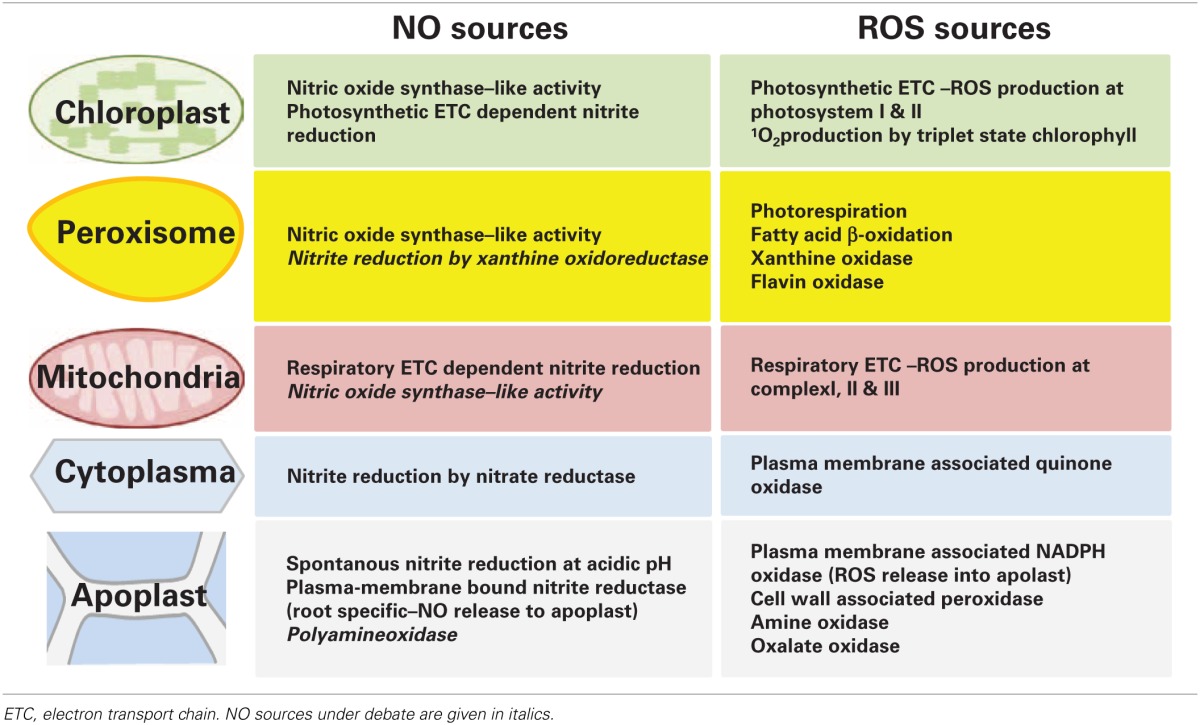
**Localization of NO and ROS sources in plant cells**.

ETC, electron transport chain. NO sources under debate are given in italics.

Cellular compartments simultaneously producing NO and ROS might be focal points of stress signaling (Table 2). While chloroplasts and mitochondria are major sources of ROS from photosynthetic and respiratory ETC these organelles are also capable of NO synthesis, one proposed mechanism being the transfer of electrons from the ETCs to NO^−^_2_ by a nitrite: NO-reductase activity. Such ETC-dependent NO formation was observed in isolated choroplasts from tobacco supplied with 25–100 μM NO^−^_2_ and in mitochondria of tobacco suspension cells under anoxia (Planchet et al., 2005; Jasid et al., 2006). More work is needed for investigating if this pathway is active also in stress responses under normoxic conditions. Mammalian NOS oxidizes arginine to citrulline and NO. Although NOS-like activity is considered the most important source of NO accumulation in plant reactions to various stresses the corresponding plant NOS still awaits identification (Leitner et al., 2009; Mur et al., 2013). Recent publications reported on the detection of a NOS-like activity in chloroplasts (Jasid et al., 2006; Tewari et al., 2013). In *A. thaliana* and *Brassica napus* protoplasts NO generation was highest immediately after the isolation procedure and decreased during culture. Experiments with a NOS activity assay and specific enzyme inhibitors suggested that NO originated from a NOS-like source. Moreover, simultaneous accumulation of NO and ROS resulted in the formation of ONOO^−^ as detected by the fluorescent dye aminophenyl fluorescein (APF) (Tewari et al., 2013). In line with this, treatment with the fungal elicitor cryptogein also triggered rapid accumulation of both NO and ROS in tobacco epidermal cells (Foissner et al., 2000). The above data imply that stress induces the accumulation of ROS and RNS in the chloroplast, which could then locally effect on photosynthesis or diffuse out of the chloroplast to other cellular compartments.

To date, there is no convincing proof of NOS-like activity in mitochondria (Table 2; Gupta et al., 2011). In contrast, peroxisomes are a source of NO both during salt stress as well as developmental processes such as lateral root growth (Corpas et al., 2009; Schlicht et al., 2013). In *A. thaliana* transgenic lines expressing GFP linked to peroxisomal targeting signal 1 (PTS1) fluorescence of the NO-specific dye diaminorhodamine co-localized with GFP fluorescence in the peroxisomes. Isolated peroxisomes displayed NOS-like activity, which was calcium dependent and could be inhibited by NOS inhibitors (Table 2). 100 mM NaCl stimulated NO synthesis in peroxisomes, which spread into the cytosol, where it probably contributed to ONOO^−^ formation and protein tyrosine nitration (Corpas et al., 2009). Peroxisomes are active sites of ROS scavenging as well as formation. The main function of peroxisomes is the removal of ROS originating from photosynthetic and mitochondrial ETCs. For this purpose, peroxisomes contain large amounts of CAT but also APX and other antioxidant enzymes. However, after a stress stimulus antioxidant enzymes can be down-regulated possibly by S-nitrosylation or nitration rendering peroxisomes a ROS source rather than a sink (Sandalio et al., 2013). Peroxisomes are often closely associated with mitochondria and/or chloroplasts. Such functional units are essential for efficient ROS scavenging but it can be speculated that they also represent “reaction vessels” for enhancing ROS/RNS signal interaction.

In the past, microscopic studies with NO-specific dyes suggested higher stress-induced NO accumulation in chloroplasts and peroxisomes than in the cytoplasm (e.g., Foissner et al., 2000; Gaupels et al., 2008; Corpas et al., 2009). One possible explanation for this finding would be that the cytoplasm has a rather low capacity of NO synthesis. While NOS-like activity was not detected, nitrate reductase (NR) is the only confirmed NO source in the cytoplasm (Table 2). However, under normal growth conditions NR preferably reduces NO^−^_3_ to NO^−^_2_, which is then further reduced by nitrite reductase to NH^+^_4_. Only under special conditions such as anoxia when NO^−^_2_ reaches high levels NR reduces NO^−^_2_ to NO at considerable rates (Gupta et al., 2011; Mur et al., 2013). For this reason, it seems unlikely that NR significantly contributes to rapid stress signaling by NO. Overall, chloroplasts and peroxisomes are probably the most important sources of NO and ROS during stress responses. Available data indicate that both signal molecules are produced simultaneously giving rise to the formation of RNS such as ONOO^−^. ROS mainly originated from NADPH oxidases and ETCs. The NO burst was driven by a yet unidentified NOS-like activity in chloroplasts and peroxisomes. Nitrite reduction to NO either non-enzymatically or by various reductases is thought to contribute comparably less to the NO burst.

## Interactions between NO and ROS

Chemical interactions between NO and ROS influence concentration, composition and signaling functions of both reaction partners. For instance, H_2_O_2_ was proposed to react with NO yielding ^1^O_2_ and NO^−^ *in vitro* (Noronha-Dutra et al., 1993). If this chemical pathway occurs *in vivo* is still ambiguous since NO is a rather stable radical, which does not easily bind non-radical species such as H_2_O_2_. Physiologically more significant is the fusion of NO with O^−^_2_ to give ONOO^−^ (Table 3) (Hill et al., 2010). This radical-radical reaction has a high rate constant and is favored instead of O^−^_2_ dismutation to H_2_O_2_. As a result, highly cytotoxic and long-lived ROS are replaced by ONOO^−^, which is short-lived in the cellular environment (Pryor et al., 2006). The exact pathway of ONOO^−^ and ONOOH (peroxynitrous acid) decay to NO^−^_2_ and NO^−^_3_ at neutral pH is still debated (Table 3). It was suggested that ONOOH isomerises to NO^−^_3_ and H^+^ either directly or indirectly via the radical intermediates NO_2_ and OH (Goldstein and Merenyi, 2008; Koppenol et al., 2012). The peroxynitrite anion on the other hand yields the RNS NO_2_, NO, and N_2_O_3_ during its degradation to NO^−^_2_ (Goldstein and Merenyi, 2008). At neutral pH ONOO^−^ and ONOOH are both present in cells and together form peroxynitrate (O_2_NOO^−^/O_2_NOOH), which decays to NO^−^_2_ and O_2_ as well as ^1^O_2_ and NO^−^ (Khan et al., 2000; Jourd'heuil et al., 2001; Gupta et al., 2009; Miyamoto et al., 2009). Meanwhile it is widely accepted that CO_2_ is an important modulator of ONOO^−^ chemistry in cells. The atmospheric gas rapidly reacts with ONOO^−^ resulting in NO^−^_3_ and the radicals NO_2_ and CO^−^_3_ (carbonate anion radical Bonini et al., 1999; Pryor et al., 2006).

**Table 3:** **Reaction stoichiometry between ROS and RNS**.

**ROS**	**RNS**
Hydrogen peroxide: H_2_O_2_	Nitric oxide: NO
Superoxide: O^−^_2_	Peroxynitrite: ONOO^−^
Singlet oxygen: ^1^O_2_	Peroxynitrous acid: ONOOH
Hydroxyl radical: OH	Peroxynitrate: O_2_NOO^−^
Oxygen: O_2_	Peroxynitric acid: O_2_NOOH
	Nitrosonium cation: NO^+^
	Nitroxyl anion:NO^−^
	Nitrogen dioxide: NO_2_
	Dinitrogentrioxide: N_2_O_3_
	Nitrosoglutathione: GSNO
**REACTION STOICHIOMETRY**	**References**
NO^−^_2_ + 2 H^+^ ↔ NO + H_2_O	Pryor et al., 2006
NO^+^ + H_2_O_2_ → ONOO^−^ + 2 H^+^	Beligni and Lamattina, 2002
NO + O^−^_2_ → ONOO^−^	Miyamoto et al., 2009
2 NO + O_2_ → 2 NO_2_	Moller et al., 2007
NO_2_ + NO ↔ N_2_O_3_	Moller et al., 2007
N_2_O_3_ + H_2_O → 2 NO^−^_2_ + 2 H^+^	Moller et al., 2007
ONOOH → ONOO^−^ + H^+^ (Ionisation)	Koppenol et al., 2012
ONOOH → NO^−^_3_ + H^+^ (Isomerisation)	Koppenol et al., 2012
ONOOH → NO_2_ + HO (Homolysis)	Koppenol et al., 2012
ONOO^−^→ NO + O^−^_2_ (Homolysis)	Koppenol et al., 2012
O_2_NOO^−^ ↔ NO_2_ + O^−^_2_(Homolysis)	Gupta et al., 2009
ONOOH + ONOO^−^ → O_2_NOO^−^+ NO^−^_2_ + H^+^	Gupta et al., 2009
CO_2_+ ONOO^−^ → CO^−^_3_ +NO_2_	Pryor et al., 2006

High levels of NO can react with O_2_ giving rise to the NO_2_ radical (Table 3). This pathway is slow in the cytosol but might be efficient in membrane-rich cellular compartments such as chloroplasts and mitochondria owing to the lipophilic nature of NO and O_2_ (Liu et al., 1998; Pryor et al., 2006). Under continuous NO production NO_2_ will further react to N_2_O_3_ (Pryor et al., 2006; Moller et al., 2007). All reactive nitrogen oxides decompose to the stable derivatives NO^−^_2_ and NO^−^_3_ within cells. However, as described in the previous section, under acidic conditions e.g., in macrophages and in the plant apoplast N_2_O_3_, NO, and NO^+^ can also originate from NO^−^_2_ upon enzymatic or non-enzymatic reduction (Table 3) (Pryor et al., 2006; Combet et al., 2010; Frohlich and Durner, 2011). Hence, dependent on the prevailing cellular environment NO and ROS can interact resulting in the formation of intermediates with distinct molecular properties. For instance, NO, NO^−^, NO^+^, and N_2_O_3_ bind to nucleophilic residues of proteins causing nitrosation (covalently bound nitroso/-NO adduct) and cysteine- as well as metal S-nitrosylation (coordinate nitrosyl/..NO adduct) (Hill et al., 2010; Fukuto and Carrington, 2011). In contrast, ONOO^−^ and the NO_2_ radical are involved in oxidation and nitration (covalently bound nitro/-NO_2_ adduct) of proteins the best studied modifications being 3-nitro-tyrosine residues (Arasimowicz-Jelonek and Floryszak-Wieczorek, 2011; Gaupels et al., 2011a; Radi, 2013). NO_2_ has less nitrating power than ONOO^−^ except with protein radicals, which result from the reaction of proteins with ROS or CO^−^_3_ radicals (Bonini et al., 1999; Pryor et al., 2006). To date, the CO^−^_3_ catalyzed binding of NO_2_ to tyrosyl residues is thought to be the major route of protein nitration.

NO-dependent protein modifications are reversible, which is important for efficient recovery of NO receptors during stress signaling. In mammalian cells, thioredoxins (TRX) denitrosylate proteins (Tada et al., 2008; Benhar et al., 2009). Recently, the central redox switch NPR1 was suggested to be denitrosylated by TRX-h-3 and -5 during incompatible *A. thaliana/P. syringae* interactions, which caused its monomerisation from oligomers, transfer into the nucleus and subsequent induction of PR genes (Tada et al., 2008). However, the exact mechanism of NPR1 regulation by S-nitrosylation and TRX is still debated (Lindermayr et al., 2010). Denitration of proteins in *A. thaliana* is probably mediated by peptide methionine sulfoxide reductase (PMSR) under normal growth conditions since *pmsr2-1* mutants displayed elevated protein nitration in the night (Bechtold et al., 2009). This enzyme reduces oxidized protein methionine residues using TRX as a co-substrate but how it can function as a denitratase is not yet resolved. Future research will uncover if additional reductases, peroxiredoxin oxidases and peroxidases such as TRX peroxidase are involved in stress signaling by NO-dependent protein modifications.

Apart from proteins many other molecules can be nitrated including lipids, fatty acids, amino acids and nucleotides (Arasimowicz-Jelonek and Floryszak-Wieczorek, 2011). Recently, 8-nitro-cGMP was uncovered as a down-stream signal of ABA, NO, and ROS in inducing stomatal closure at daytime, whereas cGMP regulated stomatal opening at night (Joudoi et al., 2013). 8-nitro-cGMP is now a prime example of how NO, ROS, and cGMP can be integrated in one signaling cascade triggering a physical response.

## NO and ROS influence each other's biosynthesis and degradation

ROS are well-known inducers of NO synthesis in various plant species, plant parts and tissues. For example, treatment with 100 μM H_2_O_2_ triggered NO synthesis in roots of *A. thaliana*, which was used in a screen for identification of mutants defective in NO accumulation. This way, the prohibitin PHB3 was uncovered as a regulatory element of ABA- and auxin-induced NO signaling (Wang et al., 2010). Moreover, H_2_O_2_ elicited a rapid NO burst in guard cells of mung bean leaves (*Phaseolus aureus*) (Lum et al., 2002) as well as NOS activity along with PCD in tobacco BY-2 cells (De Pinto et al., 2006). The interplay between ROS, NO and the antioxidant system will be discussed in more detail in the last section of this review. Exposure to ozone (O_3_) led to high ROS levels and rapid NO production in the leaves of *A. thaliana* plants (Ahlfors et al., 2009). During the O_3_ response NO acted as a signal in the onset of the hypersensitive response (HR) and in the regulation of defence-related genes thereby interacting with jasmonic acid (JA), ethylene and SA. In the phloem of *Vicia faba* NO accumulation upon treatment with 10 and 100 μM H_2_O_2_ was dependent on Ca^2+^ and NOS-like enzyme activity (Gaupels et al., 2008). Although induction of NO biosynthesis through H_2_O_2_ and Ca^2+^ is widely accepted, exact signaling cascades and enzymatic sources of NO are still not well-understood. Effects of H_2_O_2_ on NO scavenging enzymes such as GSNOR and hemoglobins were not yet investigated.

NO is not just a down-stream signal of H_2_O_2_ but was also reported to influence ROS production and degradation, which hints at complex feed-back regulation between both signal molecules. NO limits ROS accumulation for instance by inhibition of the ROS producing enzyme NADPH oxidase (Yun et al., 2011). After infection of *A. thaliana* with avirulent pathogens the elevated SNO content inhibited the NADPH oxidase isoform AtRBOHD by S-nitrosylation at Cys 890. According to the author's hypothesis this regulatory process constrains ROS accumulation and subsequent cell death progression (Yun et al., 2011). A means of enhancing antioxidant enzyme activities is the induction of the corresponding genes by NO. Accordingly, 2D-electrophoresis and Western blot analyses revealed that pre-treatment with the NO donor SNAP further increased the Al^3+^-induced protein levels and activities of APX, SOD, and GR, whereas NOS inhibitor and cPTIO suppressed both the Al^3+^ and the SNAP effect (Yang et al., 2013). Alternatively, NO could directly modify protein functions. In *Antiaris toxicaria* NO fumigation improved desiccation tolerance of recalcitrant seeds, which correlated with a decrease in H_2_O_2_ levels. The authors proposed that S-nitrosylation enhanced the activities of the antioxidant enzymes GR, APX, and DHAR by preventing their oxidation/carbonylation during desiccation (Bai et al., 2011). Moreover, in salt stressed *B. juncea* S-nitrosylation of a Fe-SOD caused an increase in its enzyme activity (Sehrawat et al., 2013).

More commonly, however, NO was associated with inhibition rather than activation of antioxidant enzymes. *In vitro*, tobacco APX and CAT were reversibly inhibited by GSNO, SNAP, and NOC-9 but irreversibly inactivated by SIN-1 (Clark et al., 2000). Inhibition of APX and CAT by NO donors was confirmed in isolated pea mitochondria, leaves of *Pelargonium peltatum* and suspension cultured cells of *A. thaliana* and *N. tabacum* (Murgia et al., 2004a; Arasimowicz-Jelonek et al., 2011b; Marti et al., 2013). SNP and SNAP were the most effective NO donors, whereas GSNO produced variable results. The chemical properties of the donors is an important issue because SNP releases NO^+^ and SIN-1 simultaneously O^−^_2_ and NO whereas most other donors deliver NO. Thus, dependent on the NO donor used and the prevailing redox conditions antioxidant enzyme activity could be affected due to oxidation, S-nitrosylation, nitrosation or nitration. Unfortunately, NO- and ROS-dependent protein modifications were not investigated in the above studies.

Any of the enzymes APX, SOD, MDHAR, DHAR, GR, and CAT was proposed to be S-nitrosylated and/or tyrosine nitrated *in vivo* in unstressed *A. thaliana*, salt-stressed citrus (*Citrus aurantium*), GSNO-treated potato or rice injected with H_2_O_2_ for eliciting cell death (Tanou et al., 2009, 2010; Fares et al., 2011; Kato et al., 2012; Lin et al., 2012). S-nitrosylation, however, was only confirmed for APX from GSNO-treated potato leaves (Kato et al., 2012). In the same study DHAR was demonstrated to be S-nitrosylated and inhibited by NO. A possible target Cys essential for enzymatic function was revealed by point mutation of candidate Cys residues. Human manganese SOD is a mitochondrial protein that undergoes site-specific nitration at Tyr34 during inflammation. Inactivation of Mn-SOD by nitration provokes oxidative stress and ultimately dysfunction of mitochondria (Radi, 2013). It would be interesting to elucidate if plant SODs are targets of nitrating species with possible roles e.g., in PCD. Collectively, the discussed data suggest that APX, CAT, and DHAR are good candidates for NO-regulated antioxidant enzymes in plants. A systematic approach is needed for deciphering, which antioxidant enzymes are controlled by NO under stress conditions, and what are the underlying molecular mechanisms.

**Figure 2:**
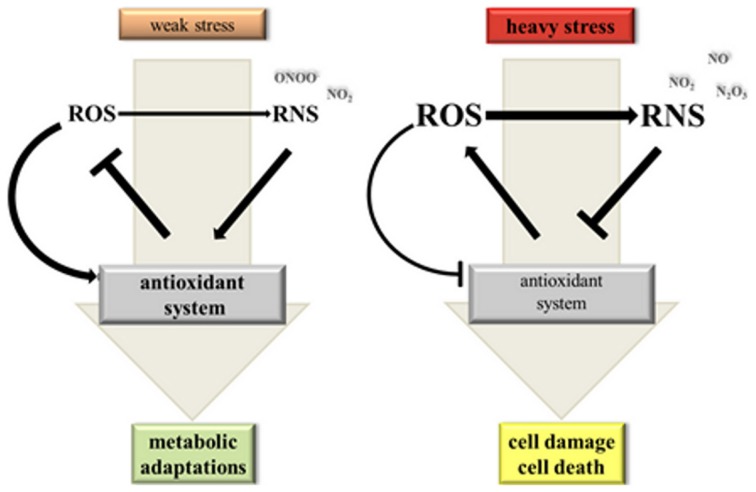
**Hypothetical model on the dynamic interaction between NO, ROS and the antioxidant system under stress conditions**. Weak stress triggers a moderate elevation of ROS (reactive oxygen species) and NO levels. ROS act as signals inducing NO synthesis and activation of the antioxidant system for improved metabolic adaptation. If ROS is produced at a somewhat higher rate than NO there would be mainly formation of oxidizing and nitrating RNS (reactive nitrogen species) imposing a weak oxidative stress to the cell. Heavy stress leads to a strong ROS and RNS burst. High NO levels promote formation of N_2_O_3_ from NO_2_ and NO and consequently nitrosative stress. Under these conditions ROS and RNS inhibit the antoxidant system causing damage and ultimately death of plant cells.

We mentioned before that NO bioactivity has been implicated both in increased as well as decreased antioxidant enzyme activities and ROS levels. One way of explaining the contradictory findings is based on the hypothesis that NO has a dose-dependent effect on the cellular redox status (Figure 2) (Thomas et al., 2008). At low concentrations NO might stimulate the antioxidant system and promote cell survival while high concentrations of NO cause severe cell damage and even death. In this model trace NO would preferably react with nucleophiles such as lipids, DNA and metal centered proteins but also with oxygen species forming oxidizing and nitrating species including ONOO^−^ and NO_2_. Little damage and NO-induced signaling will be perceived by the cell triggering antioxidant defence and repair mechanisms. Profound NO production, on the other hand, would promote secondary reactions of NO_2_ and ONOO^−^ with NO and consequently the accumulation of N_2_O_3_. This would shift conditions in the cell from weak oxidative stress toward heavy nitrosative stress, which—according to the hypothesis of Thomas et al. (2008)—inflicts severe damage ultimately leading to cell death. For some biological effects the duration of NO production is decisive because certain target molecules bind NO very slowly or need sequential NO and ROS modifications (Thomas et al., 2008). Thus, in addition to the chemical environment of the cell, which defines the RNS/ROS composition, the extent of NO production is critical in shaping stress signaling by NO.

## Interactions between NO and antioxidants

The versatility of signaling by RNS and ROS is further extended by their interaction with antioxidants. Reduced ascorbate does not react with NO but with nitrosating species NO^+^, N_2_O_3_ and with S-nitrosothiols (Scorza et al., 1997; Kytzia et al., 2006). Consequently, NO is released and AsA is converted to DHA (Combet et al., 2010). DHA spontaneously decays to the ascorbyl radical, which can combine with NO to give O-nitrosoascorbate. The latter finally undergoes hydrolysis to ascorbate and NO^−^_2_ (Kytzia et al., 2006). AsA can also scavenge ONOO^−^ with rather slow kinetics at neutral pH but rapid kinetics at pH 5.8 yielding NO^−^_2_ and NO^−^_3_ via unknown intermediates (Kurz et al., 2003). Likewise, GSH affects ONOO^−^ levels either by reduction to NO^−^_2_ or by radical-radical interactions of NO_2_ with the glutathiyl radical resulting in the formation of nitroglutathione GSNO_2_, which in turn can release NO (Balazy et al., 1998). Moreover, GSH effectively prevents ONOO^−^ mediated tyrosine nitration by re-reducing tyrosyl radicals and catalysing the formation of non-nitrating O_2_NOO^−^ from NO_2_ and O^−^_2_ (Kirsch et al., 2001). The biological significance of the above proposed pathways of ONOO^−^ degradation remains to be investigated. However, the high concentrations of GSH and AsA in plant cells could contribute to maintaining low levels of NO derivatives under non-stress conditions.

Other known plant scavengers of ONOO^−^ include gamma-tocopherol (vitamin E; Desel et al., 2007), carotenoids and the flavonoids ebselen, epicatechin and quercetin (Haenen et al., 1997). Some of the above compounds are not specific for ONOO^−^ but scavenge NO and ROS, too. Recently, cytokinins were demonstrated to be involved in controlling NO levels in *A. thaliana* (Liu et al., 2013). Continuous root-uptake of 120 μM SNP severely inhibited growth of *A. thaliana* WT plants whereas the mutant line cnu-1/amp1 was resistant to the same NO treatment. Further characterization of the mutant revealed a correlation between NO resistance and elevated cytokinin levels. Accordingly, WT plants infiltrated with the cytokinin zeatin displayed improved growth on SNP-loaded agar medium. *In vitro*, zeatin was nitrated by peroxynitrite, which produced 8-nitro-zeatin. *In vivo*, SNP caused strong accumulation of 8-nitro-zeatin in cnu-1 as compared to WT. From these results, the authors concluded that cytokinins regulate NO levels by binding the NO derivative ONOO^−^ (Liu et al., 2013).

NO interacts with glutathione in various ways. At the transcriptional level SNP and GSNO stimulated genes involved in GSH synthesis causing elevated levels of total glutathione in *Medicago truncatula* roots (Innocenti et al., 2007). Accordingly, NO donor treatment triggered an increase in total glutathione in 8 of 10 studies summarized in Table 1. In contrast, SNP had no strong effect on GSH concentrations in tobacco BY-2 cells (De Pinto et al., 2002). At the level of chemical interactions GSH binds NO by S-nitrosylation. GSNO is formed either after (1) ROS-induced accumulation of glutathiyl radicals, which bind NO with rate constants near the diffusion-controlled limit (Madej et al., 2008) or after (2) S-nitrosylation of GSH by nitrogen oxides such as NO^+^ and N_2_O_3_ (Broniowska et al., 2013). GSNO then functions as storage and transport form of NO. It is regarded as an endogenous NO donor, which releases free NO (2 GSNO → 2 NO + GSSG) or S-nitrosylates proteins by transferring the nitroso adduct (Broniowska et al., 2013; Mur et al., 2013).

## Enzymatic regulation of NO homeostasis by GSNOR, hemoglobin and pro- as well as antioxidant enzymes

**Figure 3:**
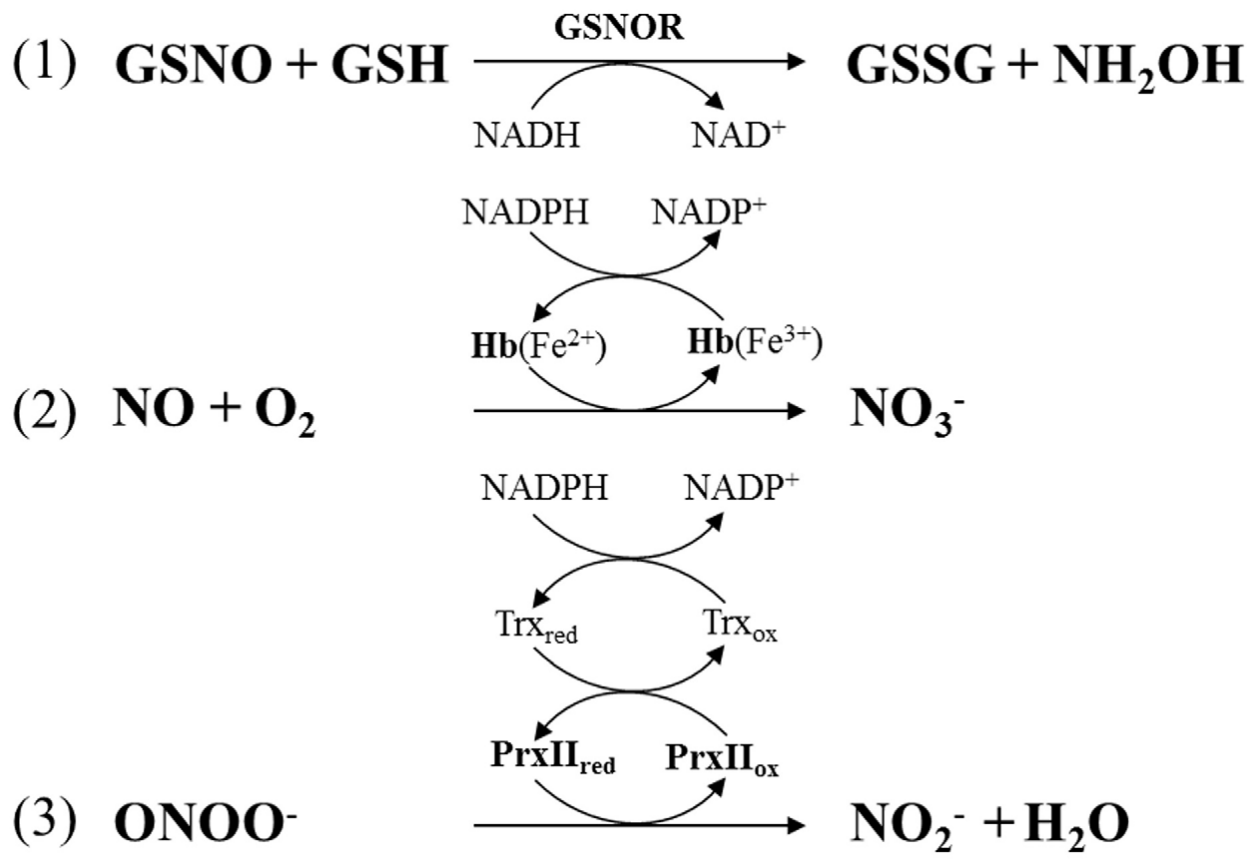
**Enzymatic regulation of NO homeostasis by (1) S-nitrosogutathione reductase (GSNOR), (2) hemoglobin (Hb), and (3) peroxiredoxin IIE (PrxIIE)**. PrxIIE is reduced by thioredoxin (Trx).

Levels of the S-nitrosylated tripeptide GSNO are tightly controlled by the enzyme GSNOR. This GSH-dependent formaldehyde dehydrogenase catalyzes the transformation of GSNO to GSSG and hydroxylamine (NH_2_NO) in the presence of GSH and NADH as the reducing species (Figure 3) (Liu et al., 2001; Sakamoto et al., 2002). In *A. thaliana* silencing or mutation of *GSNOR1* caused accumulation of S-nitrosothiols, NO and NO^−^_3_ indicating that the corresponding enzyme is a major player in NO homeostasis (Sakamoto et al., 2002). *GSNOR1* deficient plants were severely affected in growth and development (Kwon et al., 2012). They also showed increased resistance to the herbicide paraquat and altered responses toward heat stress and pathogen infection (Diaz et al., 2003; Feechan et al., 2005; Rusterucci et al., 2007; Lee et al., 2008; Chen et al., 2009; Holzmeister et al., 2011). In addition to control of NO levels, GSNOR is also indirectly involved in protein denitrosylation because GSNO and S-nitrosylated proteins are in equilibrium (Benhar et al., 2009; Malik et al., 2011). For more information on GSNOR functions refer to recent reviews (Leitner et al., 2009; Gaupels et al., 2011a; Mur et al., 2013). In mammalian/human cells CuZn-SOD and GPX (glutathione peroxidase) were proposed to use GSNO as a substrate and might act in protein denitrosylation without physiological functions being well-established yet (Benhar et al., 2009).

Another upcoming topic is the modulation of NO homeostasis by plant hemoglobins. Class-1 Hb1 catalyse the turnover of NO to NO^−^_3_ thereby influencing growth, development and stress responses (Figure 3) (Hill et al., 2010; Hebelstrup et al., 2012). Particularly, the role of alfalfa and *A. thaliana* Hb1 in hypoxia has been studied in more detail (Dordas et al., 2003; Perazzolli et al., 2004; Hebelstrup et al., 2012). It was shown that hypoxia triggered expression of the Hb1-coding gene in roots, probably for confining the stress-induced accumulation of NO. Reduced expression of *Hb1* in transgenic and mutant lines caused an increase in NO levels concomitant with decreased plant growth whereas *Hb1* over-expression improved plant fitness during hypoxia. By scavenging NO the plant might suppress a costly defence response for saving energy and valuable nitrogen under limited oxygen availability (Hebelstrup et al., 2012). Recently, Hb1 was found to be involved in pathogen resistance. *A. thaliana* mutants defective in the Hb1-coding gene *GLB1* were more resistant to the hemibiotrophic *P. syringae* and the necrotrophic fungus *Botrytis cinerea* (Mur et al., 2012). The mutant phenotype was reversed by over-expression of *GLB1* under control of the 35S promoter. The enhanced resistance in the *glb1* mutant correlated with accumulation of SA, JA, and ET. *GLB1* was down-regulated in WT plants during infection, which probably facilitated the induction of defence responses by NO accumulation.

Notably, human hemoglobin degrades ONOO^−^ to NO^−^_3_ *in vitro* further extending possible functions of hemoglobins in NO signaling (Romero et al., 2003). By comparison plants have evolved efficient mechanisms for enzymatic detoxification of ONOO^−^ by thiol-dependent peroxidases. The *A. thaliana* peroxiredoxin IIE (PrxII E) and glutathione peroxidase 5 (Gpx5) of poplar both reduce ONOO^−^ to NO^−^_2_ (Figure 3) (Sakamoto et al., 2003; Romero-Puertas et al., 2008; Ferrer-Sueta and Radi, 2009). Both enzymes are then reactivated by thioredoxin in a NADPH-consuming manner. Hence, thioredoxin functions include ROS and ONOO^−^ scavenging as well as protein denitrosylation illustrating again the essential roles of this enzyme in ROS and RNS control.

At neutral (but not acidic) pH NO^−^_2_ is a rather stable decomposition product of NO and its derivatives. However, a number of plant enzymes can convert NO^−^_2_ to RNS most prominent examples being nitrite reductase and nitrate reductase, which reduce NO^−^_2_ to NO (Stöhr et al., 2001; Morot-Gaudry-Talarmain et al., 2002; Gupta et al., 2011). During severe hypoxia deoxygenated *A. thaliana* Hb1 might act as nitrite reductase although with rather slow kinetics (Tiso et al., 2012). Given the high concentrations of NO^−^_2_ in hypoxic plant tissues Hb1 might still significantly contribute to NO accumulation (Sturms et al., 2011). A more wide-spread phenomenon could be the nitration-promoting activity of peroxidases. For instance, three *A. thaliana* hemoglobins and Hb1 of *Medicago sativa* were capable of mediating protein nitration via NO^−^_2_ oxidation to NO_2_ by a H_2_O_2_-dependent peroxidase activity (Sakamoto et al., 2004; Maassen and Hennig, 2011). Sakihama et al. (2003) demonstrated the enzymatic nitration of *p*-coumaric acid by action of horseradish peroxidase in the presence of NO^−^_2_ and H_2_O_2_. All the above data on Hb1 acting as nitrite reductase and enzymatic nitration by peroxidases were obtained *in vitro* and it is difficult to draw any meaningful conclusions for the *in vivo* situation.

## NO and redox signaling in cell death

ROS and RNS are major players in plant stress signaling. In this section we will survey current knowledge on the roles of ROS, RNS and elements of the antioxidant system in cell death events induced by biotic and abiotic stressors. Plant PCD was described as a genetically controlled cell suicide exhibiting marked similarities but also considerable differences to apoptosis in animal/human cells (Mur et al., 2008; De Pinto et al., 2012). Plants attacked by an avirulent pathogen develop HR, which is a defence mechanism for restricting the spread of pathogens by cell wall reinforcement, production of defensive secondary metabolites and ultimately cell death (Mur et al., 2008).

Almost 20 years ago Chris Lamb and his co-workers discovered that soybean cells infected with avirulent *Pseudomonas syringae pv. glycinea* accumulated high levels of H_2_O_2_, which functioned as a cell death inducer during the HR (Levine et al., 1994). Suppression of the pathogen-induced H_2_O_2_ burst by the NADPH oxidase inhibitor diphenylene iodonium (DPI) prevented cell death whereas low millimolar concentrations of exogenous H_2_O_2_ triggered HR-PCD in a calcium-dependent manner (Levine et al., 1994, 1996). Later, researchers of the same group demonstrated that NO was another essential messenger in cell death execution (Delledonne et al., 1998). Application of a NO scavenger and a NOS activity inhibitor both reduced HR-PCD of soybean suspension cells infected with avirulent bacterial pathogens. Importantly, SNP triggered cell death most efficiently in conjunction with ROS but not in the presence of DPI or CAT. ROS donors in turn efficiently killed soybean cells only if applied together with SNP (Delledonne et al., 1998). Comparable results were obtained with tobacco BY-2 cells. Simultaneous application of SNP and the H_2_O_2_-generating donor system glucose/glucose oxidase but not each individual donor alone caused a drop in ascorbate and glutathione levels, inhibition of APX and consequently PCD of tobacco BY-2 cells (De Pinto et al., 2002). Therefore, it was postulated that NO and ROS cooperate in cell death signaling (Figure 2).

Recent studies have begun to unravel the underlying modes of interactions between NO, ROS and the antioxidant system during PCD. It was shown that ONOO^−^ arose in *A. thaliana* plants challenged by avirulent *Pseudomonas syringae* (Gaupels et al., 2011b). The peak of ONOO^−^ formation from NO and O^−^_2_ coincided with the onset of the PCD. In unstressed plants ONOO^−^ was continuously scavenged by PrxIIE, which was inhibited by S-nitrosylation in course of the HR (Romero-Puertas et al., 2007). The fact that ONOO^−^ levels are controlled in a sophisticated manner would imply an important role of this RNS in the induction of cell death and pathogen resistance. However, contrary to mammalian cells this RNS does not kill plant cells (Delledonne et al., 2001). It was demonstrated that SOD, GR, CAT, and APX, which are all involved in ROS depletion, can be tyrosine nitrated by ONOO^−^ (Chaki et al., 2009; Lozano-Juste et al., 2011). If this is a significant process *in vivo* remains to be proven.

H_2_O_2_ rather than O^−^_2_ was proposed to be a pivotal signal in regulating PCD. This particular ROS acts as an inducer of NO synthesis in tobacco cells (De Pinto et al., 2006) and in mutant plants with disturbed redox homeostasis. For instance, rice knock-out mutants defective in a CAT-coding gene showed increased H_2_O_2_ levels, nitrate reductase-dependent accumulation of NO and spontaneous leaf cell death (Lin et al., 2012). Application of the NO scavenger PTIO mitigated the cell death phenotype. The importance of a down-regulation of ROS detoxifying enzymes during PCD was further corroborated by the finding that overexpression of thylakoidal APX led to a higher resistance against SNP induced cell death (Murgia et al., 2004b). In *A. thaliana* WT plants 5mM SNP triggered H_2_O_2_ accumulation and cell death, which was both reduced in the transgenic line probably because H_2_O_2_ was degraded by the elevated APX activity in these plants. The antioxidant enzymes CAT and APX control H_2_O_2_ levels under mild stress conditions. Severe cadmium stress triggered NO as well as H_2_O_2_ accumulation and senescence-like PCD of *A. thaliana* suspension cultured cells (De Michele et al., 2009). However, co-treatment with the NOS inhibitor L-NMMA prevented the NO-dependent inhibition of CAT and APX, which in turn reduced H_2_O_2_ levels and increased cell viability under cadmium stress.

Mechanical wounding provokes cell damage, which could serve as a point of entry into the plant e.g., for pathogenic bacteria. To avoid this, PCD is triggered in intact cells nearby the damaged cells for sealing the wound site. In wounded leaves of *Pelargonium peltatum* NO accumulation was restricted to the site of injury (Arasimowicz et al., 2009). Treatment with cPTIO confirmed that NO inhibited APX and CAT activity thereby temporarily enhancing the H_2_O_2_ content at the edge of the wound. Pre-treatment of leaves with NO donors before wounding prevented the H_2_O_2_ burst and reduced necrotic cell death in sweet potato (Lin et al., 2011). The exact mechanism of NO action was not determined but available data suggest that APX, GR, MDHAR and thioredoxin are S-nitrosylated during PCD, which could affect their activity (Murgia et al., 2004b; Lin et al., 2012). Inhibition of GR and MDHAR would also impact on the redox status of the glutathione and ascorbate pools. It should be considered that enzymatic activity can also be influenced by ROS-dependent modifications, which was proposed for oxidation-triggered inhibition of APX (Figure 2) (De Pinto et al., 2006). The latter enzyme was also suppressed in gene expression during PCD (De Pinto et al., 2006).

The role of NO in incompatible interactions between *A. thaliana* and avirulent *Pseudomonas syringae* was investigated using transgenic plant lines expressing a bacterial NO dioxygenase (NOD, flavohemoglobin) (Zeier et al., 2004). NOD expression attenuated the pathogen-induced NO accumulation. As a consequence the H_2_O_2_ burst was diminished and transgenic plants developed less HR-PCD and were delayed in SA-dependent *PR1* expression. These results support again the hypothesis that high levels of NO amplify redox signaling during PCD by inhibiting the plant antioxidant machinery (Zeier et al., 2004). NO and H_2_O_2_ might mutually enhance each other's accumulation by positive feed-back regulation. To this end, NO and ROS producing enzymes as well as elements of the antioxidant system must be regulated in a highly coordinate fashion for initiation of PCD. The exact signaling pathways remain to be deciphered in future studies.

However, the plant must also constrain stress signaling by NO, ROS and the antioxidant system for avoiding excessive damage by runaway cell death. Therefore, it is worth mentioning that both ROS as well as NO were found to induce genes involved in cell protection such as a gene coding for glutathione S-transferase (Levine et al., 1994). Yun and colleagues (Yun et al., 2011) even demonstrated inhibition of the ROS-producing enzyme AtRBOHD by NO in *A. thaliana* challenged by avirulent bacteria. The authors proposed a model, in which the early burst of ROS and NO initiates HR-PCD but at later stages of the defence response the SNO levels exceed a certain threshold and subsequently the AtRBOHD is inactivated by S-nitrosylation at Cys 890, which terminates the HR. In contrast to R gene-mediated resistance against avirulent pathogens, bacterial lipopolysaccharides (LPS) elicit basal pathogen resistance without onset of HR-PCD. LPS-induced NO synthesis by an arginine-dependent enzymatic source even protected plant cells against oxidative stress and cell death by enhancing the activities of CAT, SOD, and POD. The changed cellular redox status contributed to the regulation of NPR1-dependent expression of defence genes (Sun et al., 2012). In sum, NO can either act as an inducer or suppressor of plant PCD dependent on its local cellular levels and its tightly controlled interaction with ROS and elements of the antioxidant system (Figure 2).

## Concluding remarks

ROS and NO are increasingly recognized signaling molecules in plant physiology. While research on ROS has a long history NO came into focus only 15 years ago. In the present paper we reviewed recent literature dealing with the interaction between ROS, NO and the antioxidant system during stress defence. As one interesting outcome we found that exposure of plants to unfavorable conditions inevitably induced ROS but not necessarily NO accumulation. ROS can arise as a toxic by-product of disturbed energy metabolism and/or can be produced for signaling purposes. In contrast, NO is rather a highly specialized second messenger, which modifies ROS signaling or acts independently of ROS. Significantly, ROS and NO bursts are often triggered simultaneously—sometimes even in the same cellular compartment. Particularly chloroplasts and peroxisomes are hotspots of NO-ROS interactions. NO, ROS and antioxidants chemically react resulting in the formation of RNS such as ONOO^−^, NO_2_, N_2_O_3_, and GSNO. More indirect interactions include induction of NO synthesis by H_2_O_2_ and accumulation of ROS due to inhibition of antioxidant enzymes by NO-dependent protein modifications. Uncontrolled self-amplification of ROS/RNS signaling might provoke nitrosative stress and ultimately PCD. Therefore, plants have developed efficient measures for controlling NO levels by GSNOR, hemoglobins and other RNS scavenging enzymes. This review was also aimed at investigating the extreme versatility of possible reactions between NO, ROS and the antioxidant system. Many of the discussed findings originate from *in vitro* systems or animal/human models. More basic research is urgently needed for defining chemical reactions and their products actually occurring *in planta*.

### Conflict of interest statement

The authors declare that the research was conducted in the absence of any commercial or financial relationships that could be construed as a potential conflict of interest.
